# Risk factors for the poor prognosis of Benign esophageal perforation: 8-year experience

**DOI:** 10.1186/s12876-022-02624-z

**Published:** 2022-12-22

**Authors:** Qinyu Yang, Haipeng Liu, Xu Shu, Xiaoming Liu

**Affiliations:** 1 Department of Gastroenterology, Digestive Disease Hospital, The First Affiliated Hospital of Nanchang University, Nanchang, China; 2 Department of Thoracic Surgery, The First Affiliated Hospital of Nanchang University, 17 Yongwaizheng Street, Nanchang, 330006 Jiangxi China

**Keywords:** Benign esophageal perforation, Foreign body, Prognosis, Risk factor

## Abstract

**Background:**

Esophageal perforation (EP) has a high mortality rate and poor prognosis. This single-center retrospective study aims to analyze the related risk factors for benign EP.

**Methods:**

We retrospectively analyzed 135 patients with benign EP admitted to the First Affiliated Hospital of Nanchang University from January 2012 to December 2020. Univariate and multivariate logistic analysis were performed to estimate risk factors for prognosis of esophageal perforation patients.

**Results:**

There were 118 EP patients with foreign body ingestion and 17 patients with nonforeign body ingestion. Fish bones (78/118) were the most common foreign body and most of the nonforeign EPs were caused by spontaneous esophageal rupture (14/17). Foreign body perforations occurred mostly in the upper thoracic segment (70/118) and middle thoracic segment (31/118), and spontaneous esophageal ruptures occurred mostly in the lower thoracic segment (15/17). Fifteen patients (11.1%) died during hospitalization or within one month of discharge. Multivariable logistic regression analysis showed that high white blood cell (WBC) levels [odds ratio (OR) = 2.229, 95% confidential interval (CI): 0.776–6.403, *P* = 0.025], chest or mediastinal emphysema (OR = 7.609, 95% CI: 2.418–23.946, *P* = 0.001), and time to treatment > 72 h (OR = 3.407, 95% CI: 0.674–17.233, *P* = 0.018) were independent risk factors for poor prognosis.

**Conclusion:**

Foreign body was the major reason for benign EP. High WBC level, chest or mediastinal emphysema and time to treatment > 72 h were risk factors for poor prognosis.

## Introduction

Benign esophageal perforation (EP) is a life-threatening complaint in the emergency room. Nonspecific clinical symptoms often delay patient treatment, which greatly increases the risk of complications and death from esophageal injury [1].

Esophageal foreign bodies (EFBs) usually occur in young children and specific high-risk groups of adults such as those with an underlying esophageal disease, prisoners, those who are mentally retarded, and those with psychiatric illnesses, and there are multiple types and incarcerated locations of foreign bodies, which increases the difficulty of clinical treatment [2–4]. Studies have shown that foreign bodies account for 12% of all EPs, and the mortality rate is 2.1% [5]. The anatomical location of the thoracic esophagus is adjacent to the trachea, heart, and blood vessels of the chest. EFB is often accompanied by serious medical conditions, such as tracheoesophageal fistula, esophagus-aortic fistula, hemorrhage, and mediastinal abscess complications [1]. Iatrogenic causes occur as commonly as esophageal foreign bodies [6], which account for 47.6–60% of EPs [7, 8]. Another cause of nonforeign body EP is spontaneous esophageal rupture, also known as Boerhaave syndrome, which is an uncommon and life-threatening disease that was first described in 1724 [6, 9, 10]. The mortality rate associated with EP is high, between 10 and 40% reported in some centers [11–14]. Early diagnosis and treatment are considered key factor for successful treatment [15, 16].

According to the impacted position of the EFB, it can be divided into cervical, thoracic, and abdominal foreign bodies, and foreign bodies in the corresponding positions cause corresponding esophageal injury. A systematic review of the literature included 61 studies and revealed 5044 EFBs impacted in the cervical esophagus (66.9%), 1862 in the thoracic esophagus (24.7%) and 635 in the lower esophagus (8.4%) [1]. Some scholars divided EP patients into a ≤ 24 h group and > 24 h group according to whether the treatment time after eating foreign bodies exceeded 24 h [17]. In 2009, a research group from the University of Pittsburgh proposed an EP severity scoring system (PSS) based on clinical factors [11]. This scoring system ranks esophageal perforation as low, intermediate, and high risk (on a scale of 0–18) according to the patient’s clinical risk factors, and is designed to measure the severity of esophageal rupture by weighting clinical variables. Their study showed that among patients with scores ≤ 2, 3–5, and > 5, the complication rates were 53%, 65%, and 81% respectively, and the mortality rates were 1%, 3%, and 27% respectively [11]. The classification of all types is mainly based on the patient’s medical history and clinical manifestations, to guide follow-up treatment and predict prognosis, but there is no clear consensus on the classification standards.

Currently, there is no consensus on the management of benign EP. Nonoperative management included conservative and endoscopic interventions. Various surgical approaches to the treatment of esophageal perforation have been the mainstays of therapy for decades [18–20]. Although operative management remains the standard in most of patients with EP, nonoperative management may be successfully implemented in selected patients with low morbidity and mortality if favorable radiographic and clinical characteristics are present.

The treatment of benign EP generally requires individualized attention such as supportive care that needs to be further practiced and summarized in clinical practice. Although there are various treatment methods, many factors are still considered to be associated with poor prognoses, such as time to treatment, site of perforation, vital signs, and sepsis [14, 15, 21]. In this study, we analyzed the diagnosis and treatment of benign EP and determined the patients’ potential risk factors associated with poor prognosis to guide clinical treatment.

## Materials and methods

### Subjects and study design

This retrospective study was conducted in the First Affiliated Hospital of Nanchang University, and patient diagnoses of EP from January 2012 to December 2020 were retrospectively analyzed through the electronic medical record system. Diagnostic criteria were as follows: (1) With or without a history of ingestion or eating the suspicious foreign body, or fever, pain in swallowing, chest and back pain, dysphagia, foreign body sensation, and other related symptoms. (2) Lesions penetrating the outer wall of the esophagus, extraluminal air or fluid surrounding the esophagus or within the mediastinum, or pleural effusions, and other lesions detected by computed tomography (CT), endoscopy, X-ray, gastrointestinal angiography, and other auxiliary examinations.

Detailed inclusion criteria were as follows: (1) Patients diagnosed with benign EP or esophageal rupture. (2) Patients whose lesions were adjacent to intrathoracic blood vessels, important organs, mediastinum, or diseases that were within the scope of thoracic surgery due to perforation. Exclusion criteria were as follows: (1) Patients with atypical esophageal perforation or perforation and rupture not reaching the full thickness of the esophagus. (2) Patients with incomplete demographic data.

**Fig. 1:**
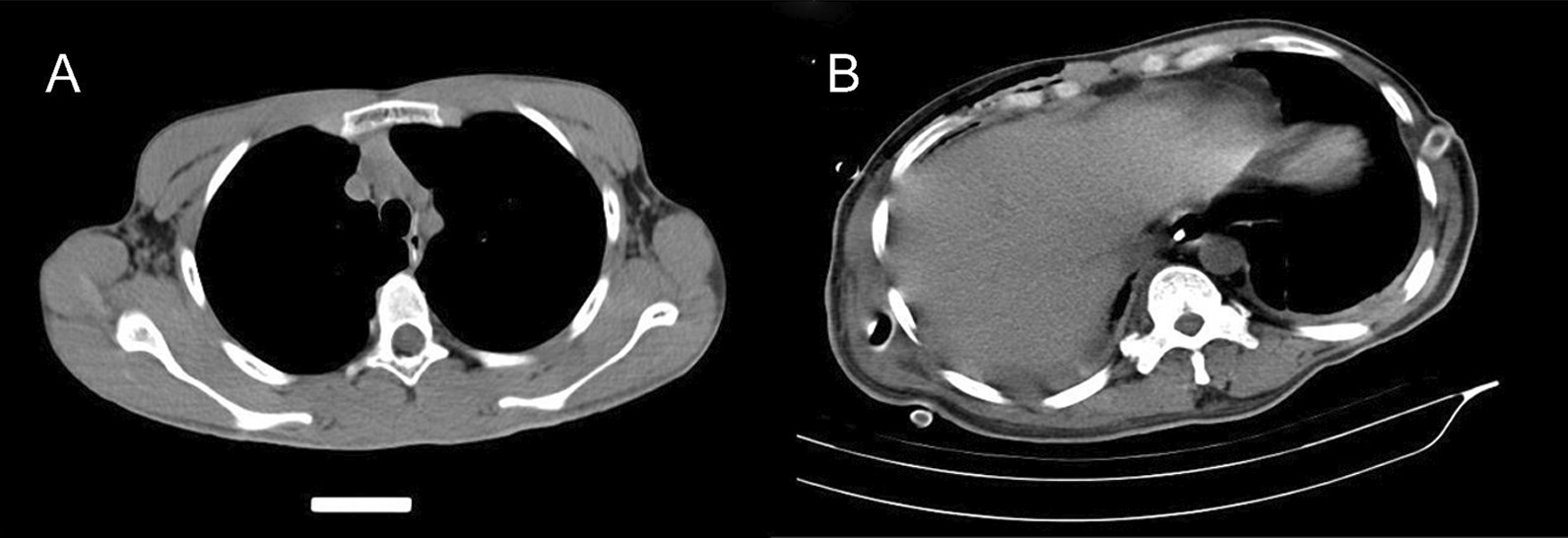
Computed tomographic images. **A** Esophageal perforation caused by foreign body. **B** Esophageal fistula

**Fig. 2:**
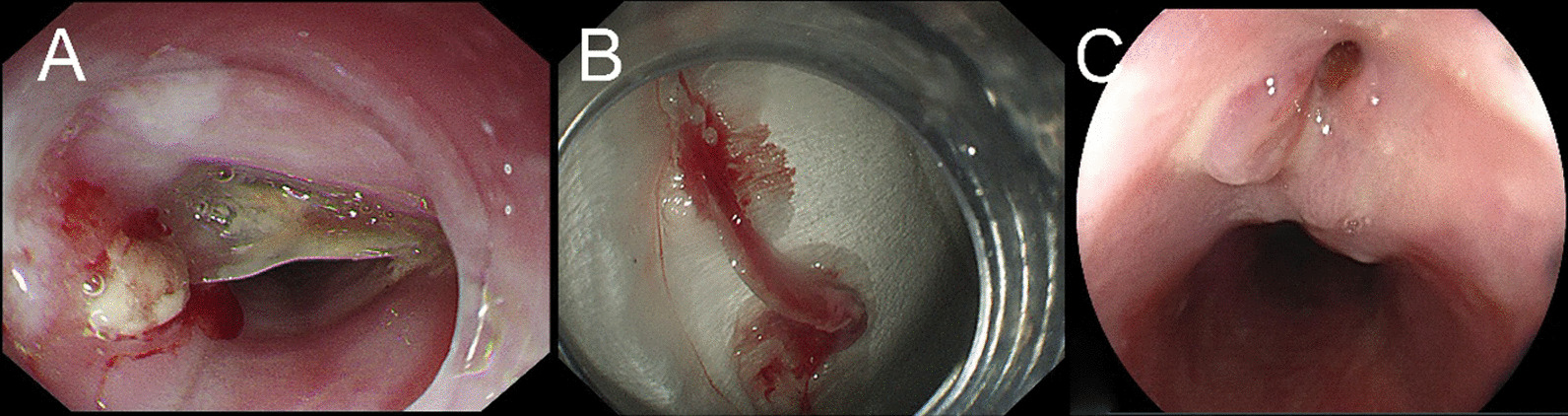
Endoscopic images. **A** Endoscopy shows a fish bone incarcerated in the esophagus. **B** The foreign body was removed. **C** Esophageal fistula

All patients with suspected perforation subsequently underwent CT (Fig. 1A). Operative management included any kind of surgical intervention. Nonoperative management comprised all nonsurgical approaches ranging from simple conservative treatment to advanced interventional and endoscopic measures such as stent insertion and removal of foreign bodies (Fig. 2A, B). We used white blood cells (WBCs) > 10.02 × 10^9^/L as the cutoff for high WBC levels. Fistula were detected by endoscopy, X-ray, CT, and other imaging methods (Figs. 1B and 2C).

The patients were divided into Groups A and B according to the prognosis. Group A: patients were in good states and no complications occurred during hospitalization or within one month of discharge. Group B: included patients who died during hospitalization or accompanied with esophageal fistula or esophagotracheal fistula during hospitalization or within one month of discharge.

### Data collection

The following information regarding initial presentation, treatment, clinical course, and outcome was collected: age, sex, etiology of perforation, symptoms, time to treatment, type of management, site of perforation, complications, hospitalization, outcome, and levels of WBCs and neutrophils before treatment and follow-up. For patients with perforation of the foreign body, time to diagnosis, time to treatment, and the situation of foreign bodies were collected. Follow-up was conducted one month after discharge.

### Statistical analysis

Statistical analyses to identify prediction factors were performed using SPSS 25.0 for Windows (SPSS, Chicago, IL). Continuous variables are expressed as the mean ± standard deviation, and categorical data are presented as absolute numbers and percentages. The chi-square test was used to determine the significance of differences between categorical variables, and the t-tests was used for continuous variables. We performed univariate logistic hazard regression analysis in a forward stepwise manner. Significant variables in univariate analysis (*P* < 0.05) were carried into a multivariate logistic analysis to obtain the odds ratio (OR) and corresponding 95% confidential interval (CI) for every independent prognostic variable. Statistical significance was designated by *P* < 0.05.

## Results

### Baseline characteristics

**Fig. 3:**
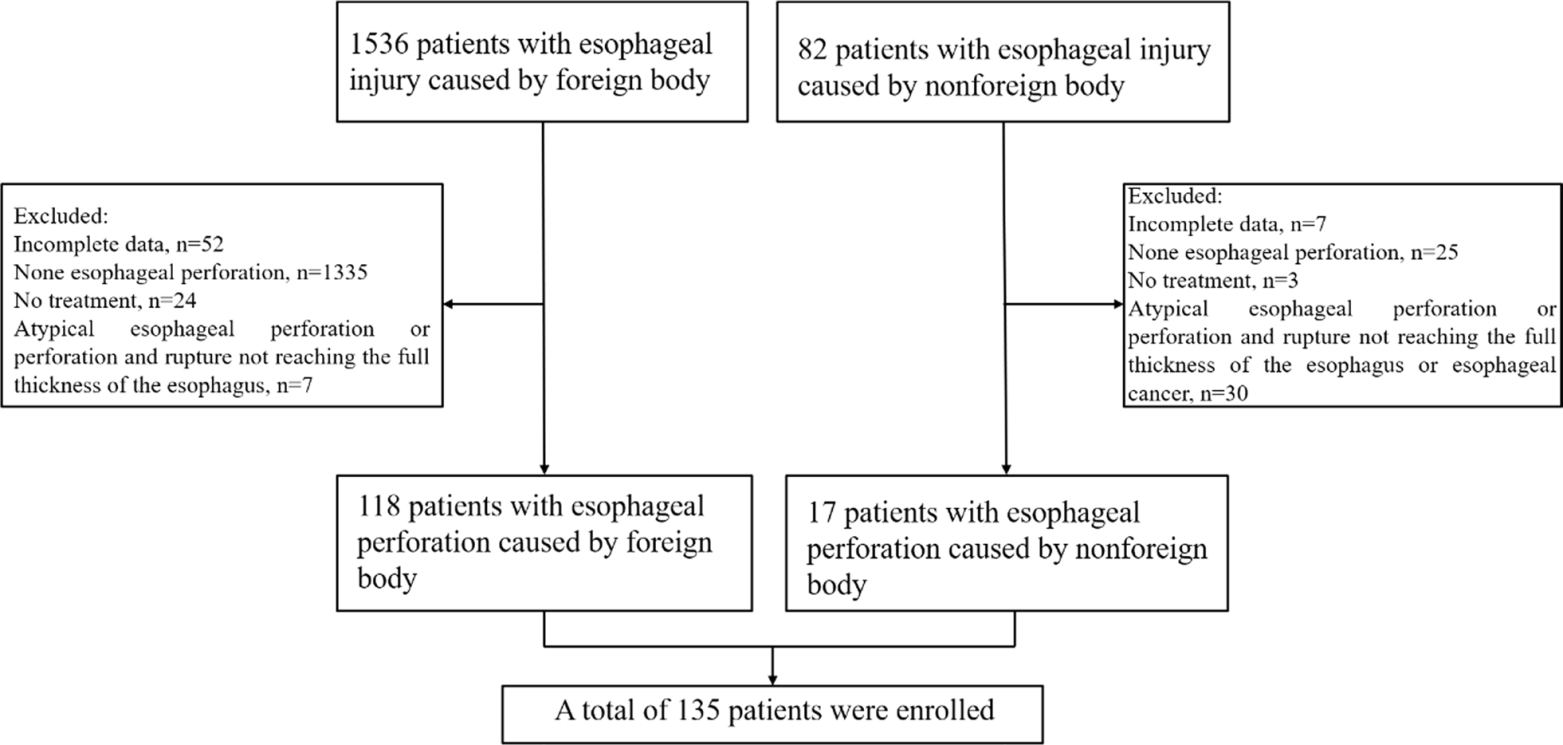
Flowchart shows the inclusion of esophageal perforation cases

**Fig. 4:**
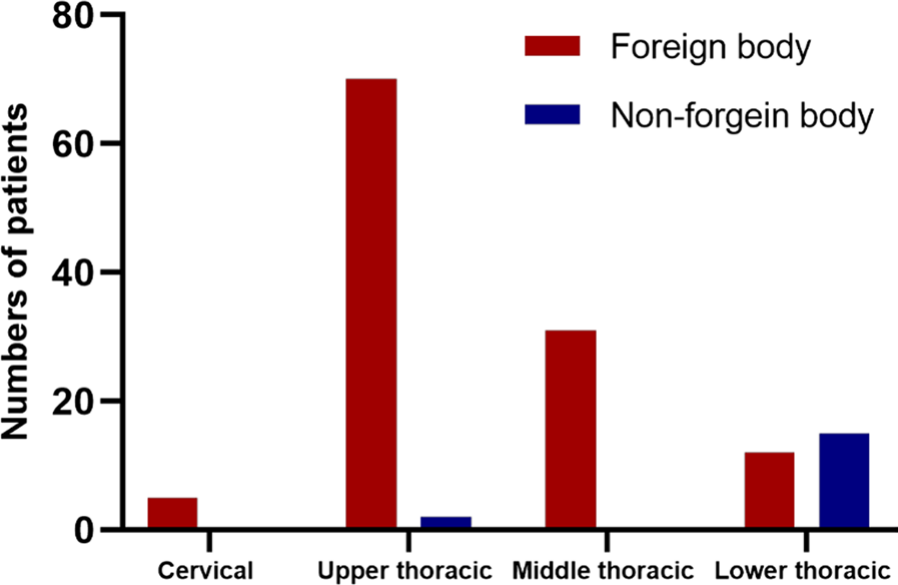
Distribution of esophageal perforation

The study comprises a total of 135 patients with EP (Fig. 3). The baseline characteristics of the patients are presented in Table 1. The mean age of these patients was 55.6 ± 14.3 years, and 77 of these patients (57.0%) were female. There were 118 cases of EP caused by foreign bodies and 17 cases developed nonforeign body perforation. The most common clinical symptoms were chest and back pain (77.8%), followed by swallowing pain (74.1%). In the foreign body group, swallowing pain was the most common symptom (73.7%), followed by chest and back pain (66.7%). Of 80 patients who received treatment for perforation after 24 h, only 40.7% (55/135) received treatment within 24 h. Sixty-eight patients (50.4%) had pleural or mediastinal pneumonitis, 46 patients (34.1%) had pleural effusion, 77 patients (57.0%) were complicated by esophageal inflammation or mediastinal abscess, and 9 patients (6.6%) developed mediastinitis. The average number of WBCs in the foreign body group was 10.1 ± 4.1 × 10^9^/L, while the average number of WBCs in the nonforeign body group was 13.6 ± 5.6 × 10^9^/L. In terms of imaging characteristics, among the patients with foreign body perforation, 84 patients (71.2%) had foreign bodies adjacent to the great intrathoracic blood vessels and pericardium, six patients penetrated the esophagus and pierced the aorta, two patients had tracheal rupture, and one patient pierced the pericardium. Two patients were misdiagnosed with esophageal tumors by CT scan, jujube pits were then detected through endoscopy. Figure 4 compares the location of the EP for different reasons. There were 70 patients (59.3%) with foreign bodies located in the upper thoracic segment, followed by 31 patients (26.3%) with foreign bodies in the middle thoracic segment. Fifteen patients (88.2%) had nonforeign body perforations located in the lower thoracic segment.

**Table 1:** Comparison between foreign body and non-foreign body group

Variable	Total cases (n = 135)	Foreign body (n = 118)	Non-foreign body (n = 17)	*P*
Sex				0.494
Male	77 (57.0)	66 (55.9)	11 (64.7)	
Female	58 (43.0)	52 (44.1)	6 (35.3)	
Age, year, mean ± SD	55.6 ± 14.3	55.6 ± 14.6	56.0 ± 11.6	0.976
Symptoms, n (%)				
Fever	33 (24.4)	27 (22.9)	6 (35.3)	0.417
Tachycardia	32 (23.7)	21(17.8)	11 (64.7)	< 0.001
Hypotension	5 (3.7)	2 (1.7)	3 (17.6)	0.010
Swallowing pain	100 (74.1)	87 (73.7)	13 (76.5)	1.000
Neck pain	14 (10.4)	12 (10.2)	2 (11.8)	1.000
Chest or back pain	105 (77.8)	90 (66.7)	15 (88.2)	0.425
Breathing restriction	32 (23.7)	17 (14.4)	15 (88.2)	< 0.001
Haematemesis	19 (14.1)	6 (5.1)	13 (76.5)	< 0.001
Time to treatment, n (%)				0.221
≤ 24 h	55 (40.7)	46 (39.0)	9 (52.9)	
> 24 h	80 (59.3)	72 (61.0)	8 (47.1)	
Chest or mediastinal emphysema	68 (50.4)	51 (37.8)	17 (100.0)	< 0.001
Hydrothorax	46 (34.1)	29 (24.6)	17 (100.0)	< 0.001
Esophagitis or esophageal abscess	77 (57.0)	63 (53.4)	14 (82.4)	0.024
WBC(×10^9^/L), mean ± SD	10.6 ± 4.4	10.1 ± 4.1	13.6 ± 5.6	0.002
Death rate, n (%)	15 (11.1)	8 (6.8)	7 (41.2)	< 0.001

WBC, white blood cell, SD, standard deviation

Types of foreign body and nonforeign body EPs are shown in Table 2. Among the foreign body group, 78 cases (66.1%) were due to fish bones, 25 cases (21.2%) were due to animal bones (chicken, duck, pig), six cases (5.1%) were due to jujube pits, other animal bones (rabbit, bullfrog, turtle, etc.) and other foreign bodies (iron pieces, braces, eggshells, etc.) had three cases each. Among the foreign body group, three patients had a history of foreign body swallowing, but no foreign body was found by intraoperative digestive endoscopy. Among the nonforeign body group, 14 patients (82.4%) had a spontaneous EP, and iatrogenic injury accounted for 11.8%. One patient developed traumatic EP.

**Table 2:** Different injury reason of esophageal perforation

Foreign body (n = 118)	N (%)	Non-foreign body (n = 17)	N (%)
Fishbone	78 (66.1%)	Spontaneous	14 (82.4%)
Animal bone*	25 (21.2%)	Iatrogenic	2 (11.8%)
Jujube pit	6 (5.1%)	Traumatic	1 (5.8%)
Other animal bone^†^	3 (2.5%)	–	–
Others^#^	3 (2.5%)	–	–
No foreign body^⊿^	3 (2.5%)	–	–

*chicken, duck, pig, etc. ^†^Rabbit, bullfrog, soft-shelled turtle, etc. ^#^iron pieces, braces, egg shells, etc. ^⊿^Patients with history of swallowing foreign body, but digestive endoscopy only showed esophageal perforation without detecting foreign body

### Treatment method and treatment result

The details the treatment methods, treatment outcomes, and causes of death are shown in Table 3. A total of 135 patients were included. Endoscopy is a major treatment for removing EFBs. Surgery is considered a last resort and is usually reserved for high-risk cases in which severe complications are suspected. During hospitalization and within one month after discharge, 15 patients (11.1%) died, 9 patients developed esophageal fistula and empyema after discharge, and 21 patients had EP wounds exceeding 1 cm.

**Table 3:** Treatment method and the result of esophageal injury

Treatment regiment	N	Death	Prognosis and complication
Gastroscopy	87	0	2 patients developed esophageal fistula
Gastroscopy + titanium clip closed	3	0	none
Gastroscopy + closed thoracic drainage	1	0	none
Gastroscopy + endovascular graft exclusion	1	0	none
Video-assisted thoracoscopic surgery vats	4	1	1 patient died of postoperative respiratory and cardiac arrest
Gastroscopy + open surgery	6	1	1 patient died of AEF, and 2 patients developed esophageal fistula
Open surgery	19	6	1 patient died of AEF, 1 patient died of respiratory failure, 3 patients died of MODS, 1 patient died of septic shock, 2 patients developed esophageal fistula, 1 patient developed pyothorax
Closed thoracic drainage	2	1	1 patient died of MODS
Gastroscopy	6	3	1 patient died of septic shock, 1 patient died of AEF, 1 patient died of respiratory failure, 1 patient developed tracheoesophageal fistula, 1 patient developed esophageal fistula
Without invasive treatment	6	3	1 patient died of aortoclasia, 1 patient died of septic shock with respiratory failure, 1 patient died of septic shock

MODS, multiple organ dysfunction syndromes, AEF, aortoesophageal fistula

### Clinical features and risk factors for prognosis of esophageal perforation

To stratify the clinical risks associated with EP, all the patients were divided into two groups according to prognosis during hospitalization or within one month of discharge. Group A: Patients were in good states and no complications occurred during hospitalization or within one month of discharge. Group B: included patients who died during hospitalization or had esophageal fistula or esophagotracheal fistula during hospitalization or within one month of discharge. As shown in Table 4, EP caused by foreign bodies usually had a poor prognosis compared to the nonforeign body group (*P* = 0.029). Patients with infection symptoms such as esophagitis or esophageal abscess (*P* = 0.003), high WBC level (*P* = 0.013), and chest or mediastinal emphysema (*P* = 0.002) tended to develop a worse outcome. There were no significant differences between Group A and Group B for age, sex, and location of perforation. The heart rate (*P* < 0.001) and temperature (*P* = 0.014) were higher in Group B than in Group A.

**Table 4:** Comparison between good and poor prognosis group

Variable	Group A, n (%)	Group B, n (%)	*P*
Total	105	30	
Age, year, mean ± SD	54.09 ± 14.29	61.1 ± 12.96	0.205
Sex			0.680
Male	61 (58.0)	16 (53.3)	
Female	44 (42.0)	14 (46.7)	
Reason of injury			**0.029**
Foreign body	95 (90.4)	22 (73.3)	
Non-foreign body	10 (9.6)	8 (26.7)	
Esophagitis or esophageal abscess	51 (48.5)	24 (80.0)	**0.003**
WBC (×10^9^/L)			**0.013**
≤ 10.02	59 (56.1)	9 (30.0)	
>10.02	46 (43.9)	21 (70.0)	
Chest or mediastinal emphysema	45 (42.8)	23 (76.6)	**0.002**
Hydrothorax	23 (21.9)	23 (76.6)	**0.000**
Heart rate (/min), mean ± SD	85.95 ± 16.73	94.7 ± 19.98	
Temperature (°C), mean ± SD	36.67 ± 0.58	37.18 ± 0.86	**0.014**
Location of perforation			0.127
Cervical	3 (2.8)	2 (6.6)	
Upper thoracic	62 (59.0)	10 (33.3)	
Middle thoracic	24 (22.8)	7 (23.3)	
Lower thoracic	16 (15.4)	11 (36.6)	
Time to treatment			**0.018**
≤ 24 h	33 (31.4)	4 (13.3)	
24–72 h	49 (46.6)	15 (50.0)	
> 72 h	23 (22.0)	11 (36.7)	

*P* value < 0.05 are shown in bold

WBC, white blood cell, SD, standard deviation

The risk factors for prognosis are provided in Table 5. Univariate analysis showed that nonforeign body, WBC > 10.02 × 10^9^/L, esophagitis or esophageal abscess, hydrothorax, chest or mediastinal emphysema and time to treatment > 72 h were significantly correlated with poor prognosis (*P* < 0.05). Multivariable logistic regression analysis showed that high WBC level (OR = 2.229, 95% CI: 0.776–6.403, *P* = 0.025), chest or mediastinal emphysema (OR = 7.609, 95% CI: 2.418–23.946, *P* = 0.001), and time to treatment > 72 h (OR = 3.407, 95% CI: 0.674–17.233, *P* = 0.018,) were independent risk factors for poor prognosis.

**Table 5:** Univariate and multivariate analysis of risk factors for prognosis of esophageal perforation patients

Variable	Univariate analysis	*P*	Multivariate analysis	*P*
Age(years)				
≤ 60	–	–	–	–
> 60	1.763 (0.777–3.999)	0.175	–	–
Gender				
Male	–	–	–	–
Female	1.213 (0.537–2.741)	0.640	–	–
Reason of injury				
Foreign body	–	–	–	–
Non-foreign body	3.455 (1.222–9.763)	**0.019**	0.721 (0.173–3.013)	0.721
WBC (×10^9^/L)				
≤ 10.02	–	–	–	–
>10.02	2.993 (1.243–7.149)	**0.014**	2.229 (0.776–6.403)	**0.025**
Esophagitis or esophageal abscess				
No	–	–	–	–
Yes	4.235 (1.601–11.206)	**0.004**	1.706 (0.526–5.535)	0.374
Hydrothorax				
No	–	–	–	–
Yes	11.714 (4.467–30.723)	**0.000**	2.013 (0.622–6.513)	0.243
Chest or mediastinal emphysema				
No	–	–	–	–
Yes	4.381 (1.728–11.105)	**0.002**	7.609 (2.418–23.946)	**0.001**
Location of perforation				
Cervical	–	–	–	–
Upper thoracic	0.242 (0.036–1.633)	0.145	–	–
Middle thoracic	0.438 (0.061–3.160)	0.413	–	–
Lower thoracic	1.031 (0.147–7.226)	0.975	–	–
Time to treatment				
≤ 24 h	–	–	–	–
24–72 h	2.534 (1.064–7.337)	0.088	–	–
> 72 h	4.351 (1.197–12.234)	**0.010**	3.407 (0.674–17.233)	**0.018**

*P* value < 0.05 are shown in bold

WBC, white blood cell

## Discussion

EP is a clinically critical disease with high mortality. Improper or delayed treatment can lead to various complications [10]. Symptoms (pain, vomiting, hematemesis, dysphagia, or tachypnea) and signs (tachycardia, fever, subcutaneous emphysema, cardiac crunch, chest personality, or dullness) of EP vary with cause and location (cervical, thoracic, or abdominal). Pain was the most common symptom, present in 70 to 90% of patients, usually referring directly to the site of perforation [22]. In our study, regardless of the cause of EP, two patients were misdiagnosed as aortic dissection due to severe chest and back pain at the first diagnosis. This suggests that in patients with relevant medical history, attention should be given to the identification of chest and back pain-related diseases. In patients with foreign body perforation, swallowing pain and chest and back pain were the most common causes due to foreign body incarceration, and tachycardia, respiratory limitation, hypotension, and other emergencies occurred less frequently. In a small number of cases, due to prolonged incarceration of the foreign body, abscesses often lead to fever, pleural effusion, and chest tightness. At the same time, this study also found that the level of WBCs in the nonforeign body group was higher than that in the foreign body group, which may indicate a heavier infection. In addition, some patients required mechanical ventilation upon admission, and early surgical debridement, and treatment for such patients can effectively reduce the complications of patients and save their lives. A study conducted by Shaker et al. [23] confirmed that early diagnosis and management (golden 24 h) were crucial for successful outcomes in patients with a ruptured esophagus.

Various reasons lead to EP. In our study, foreign bodies accounted for most benign EPs, followed by spontaneous esophageal perforation, which differs from previous studies [24, 25]. In our study, we found that foreign body EP was more common in the upper and middle thoracic segments, consistent with previous studies [26, 27], which is mostly due to the second esophagus stenosis, where the aortic arch and the left main bronchus cross, located in the upper and middle thoracic segments. At the same time, we found that spontaneous EP was almost always located in the lower thoracic segment, similar to the study conducted by Schweigert et al. [28].

Over the last two decades, advances in endoscopic technologies have facilitated salvage of the native esophagus in the setting of a perforation [1, 29]. Before treatment, physicians from the department of cardiothoracic surgery, otolaryngology, or digestive endoscopy were consulted to evaluate the patient’s clinical condition. When it is difficult to decide the operation method or there is a risk of endoscopic treatment, the surgeon should accompany the patients, and endoscopic removal under sedation or general anesthesia in the operating room should be considered. Laryngoscopy and digestive endoscopy are both diagnostic and therapeutic measures. Esophagoscopy is a traditional method to remove foreign bodies, with the advantage of protecting the airway. After endoscopic removal of the foreign body, if there is a large mediastinal abscess, a large esophageal fistula, or other conditions that require surgical treatment, early surgical treatment is generally performed. The surgical methods included abscess incision and drainage, foreign body removal, esophageal fistula repair, esophageal fistula exclusion, and secondary suturing. A previous study showed that surgical treatment is usually associated with low mortality [30]. However, in our study, the mortality rate between open surgery and thoracoscopic surgery was 27.6%. This is probably due to the presence of foreign bodies and abscesses involving or adjacent to the aorta.

At present, various studies are focusing on the risk factors affecting the prognosis of patients with benign EP. In this study, we used death combined with adverse events as treatment outcome indicators, consistent with previous studies [18, 31]. According to univariate analysis, nonforeign body, WBC > 10.02 × 10^9^/L, esophagitis or esophageal abscess, hydrothorax, chest or mediastinal emphysema, and time to treatment > 72 h were significantly correlated with poor prognosis. Multivariate analysis showed that high WBC level, chest or mediastinal emphysema, time to treatment > 72 h were prognostic factors. Various studies divided time to treatment into two types, ≤ 24 h and > 24 h, and mentioned that greater than 24 h between diagnosis and treatment served as a significant predictor [32–34]. Our study provided a more refined classification and demonstrated that time to treatment > 72 h was associated with a higher risk of poor prognosis. Studies conducted by Bhatia et al. [18] and Huang et al. [31] also, suggest that inflammation and abscess are key factors affecting patient prognosis and mortality. The scoring criteria for EP proposed by Abbas et al. [18] included pleural effusion and increased WBC counts. The criteria also included indicators such as fever, respiratory function involvement, hypotension, and obvious perforation and leakage, which were consistent with the results of our univariate analysis.

This study does have some limitations. First, our study was retrospective in a single center and had a limited sample size. Second, it only involved patients from one region, so the eating habits of these patients only reflect southern Chinese customs. Finally, the currently accepted evaluation standard for EP is the PSS, which was not evaluated in this study. In subsequent studies, the PSS should be evaluated, and its validity can be verified.

## Conclusion

Benign EP has a high mortality rate and a high risk of poor prognosis, especially for patients caused by nonforeign body. High WBC level, chest or mediastinal emphysema, and time to treatment > 72 h were independent risk factors for benign EP and were significantly associated with poor prognosis. Prompt identification of perforation as the cause of a patient’s problem, reduce the risk of infection, and timely choice of a therapeutic approach are keys to maximize successful outcomes. The results of this study may help better identify patients with EP who are at higher risk for developing poor prognoses and improve their follow-up treatment and clinical course, but large prospective randomized clinical trials are still needed.

## Data Availability

The data that support the findings of this study are available from the correspond author but restrictions apply to the availability of these data, which were used under license for the current study, and so are not publicly available. Data are however available from the authors upon reasonable request and with permission of the correspond author.

## Abbreviations

EP: Esophageal perforation
EFBs: Esophageal foreign bodies
PSS: Pittsburgh severity scoring system
CT: Computed tomography
WBCs: White blood cells
OR: Odds ratio
CI: Confidential interval
